# Improved Sensitivity MEMS Cantilever Sensor for Terahertz Photoacoustic Spectroscopy

**DOI:** 10.3390/s16020251

**Published:** 2016-02-19

**Authors:** Ronald A. Coutu, Ivan R. Medvedev, Douglas T. Petkie

**Affiliations:** 1 Department of Electrical and Computer Engineering, Air Force Institute of Technology, 2950 Hobson Way, Wright-Patterson AFB, Dayton, OH 45433, USA; 2 Department of Physics, Wright State University, 3640 Colonel Glenn Highway, Dayton, OH 45435, USA; ivan.medvedev@wright.edu (I.R.M.); doug.petkie@wright.edu (D.T.P.)

**Keywords:** MEMS, cantilever, terahertz, photoacoustic

## Abstract

In this paper, a microelectromechanical system (MEMS) cantilever sensor was designed, modeled and fabricated to measure the terahertz (THz) radiation induced photoacoustic (PA) response of gases under low vacuum conditions. This work vastly improves cantilever sensitivity over previous efforts, by reducing internal beam stresses, minimizing out of plane beam curvature and optimizing beam damping. In addition, fabrication yield was improved by approximately 50% by filleting the cantilever’s anchor and free end to help reduce high stress areas that occurred during device fabrication and processing. All of the cantilever sensors were fabricated using silicon-on-insulator (SOI) wafers and tested in a custom built, low-volume, vacuum chamber. The resulting cantilever sensors exhibited improved signal to noise ratios, sensitivities and normalized noise equivalent absorption (NNEA) coefficients of approximately 4.28 × 10^−10^ cm^−1^·WHz^−1/2^. This reported NNEA represents approximately a 70% improvement over previously fabricated and tested SOI cantilever sensors for THz PA spectroscopy.

## 1. Introduction

Chemical sensing and spectroscopic detection of chemical species can be made through various means. In this work, a direct sensing technique was performed by measuring the photoacoustic (PA) response of a microelectromechanical system (MEMS) cantilever when a gaseous sample was excited with a terahertz (THz) radiation source. The “energy deposited in the sample is measured directly [1] through the PA effect and is proportional to the radiation source power. The photoacoustic effect was first published by Alexander Graham Bell in 1880 when he found that modulated sunlight incident on a thin disk generated sound waves [2]; and since its discovery, the PA effect has been used to study solid, liquid, and gaseous states of matter. Many different sensing techniques have been employed, over the last several decades, for photoacoustic chemical sensing of trace gases and molecular analysis including: tuning forks [3,4,5,6], membrane microphones [7,8], bridges [9] and cantilevers [10,11,12,13,14,15,16,17,18].

Photoacoustic detection of radiation is an experimental technique widely used for molecular spectral detection in solids and gasses [1,11]. In this research, vibrating MEMS cantilevers were designed, modeled and fabricated, using silicon on insulator (SOI) wafers, for sensing the PA response due to sub-millimeter/terahertz radiation. The photoacoustic effect results when energy from an electromagnetic wave is absorbed by molecules and then transferred through collisions and other non-radiative pathways into translational energy [10]. When the THz radiation source is properly modulated and sufficient energy is absorbed by the gaseous species, an acoustic wave is generated that can be detected by a pressure sensitive micro-device such as a vibrating cantilever beam [15]. This research extends our previous efforts by improving the MEMS cantilever sensor fabrication to improve sensitivity through enhanced damping at very low chamber pressures [19]. Specifically, system sensitivity is improved by reducing internal beam stresses, minimizing out of plane beam curvature, optimizing beam damping and by filleting the cantilever’s anchor and free end to improve fabrication yield.

The resulting minimum sensitivity of the cantilever sensor is expressed as

(1) $$\propto_{min}=\frac{\propto_{peak}}{SNR}$$

where, *α_peak_* is the measured absorption coefficient and SNR is the measured signal to noise ratio.

Since PA systems, however, can vary widely it is important to use standardized sensitivity values when comparing results. The normalized noise equivalent absorption (NNEA) coefficient is defined as

(2) $$\text{NNEA}=\propto_{min}P_{0}\sqrt{T}$$

where, *P*_0_ is the radiation power and T is the signal averaging time.

The gap distance that defines the cantilever’s edge is critical in minimizing gas leakage around the beam and also proper beam damping during actuation. The membrane gap distance controls system damping of the system. Too much gap results in excessive gas leakage around the beam and the cantilever behaves like an undamped structure that no longer functions optimally for sensing the PA effect in low vacuum environments. This gap distance is highly dependent on the minimum feature size of the photolithography during fabrication. Additionally, the quality of the release etch to define the cantilever’s gap distant, plays an important role in device operation. Sievila* et al.* used a tetramethylammonium hydroxide (TMAH) anisotropic wet etch release process, resulting in large gaps at the cantilever’s free end corners due to undercutting at the convex corners [20,21]. The rounded off beam corners, due to isotropic wet etching, was a source of gas leakage and loss of PA system sensitivity. Sievila* et al.*’s cantilever sensor exhibited an asymmetric gap around the beam that increased at the corners from approximately 3 μm along the beam’s edge to approximately 20 μm at the corner [17].

In addition, MEMS SOI cantilevers are generally not perfectly flat due to internal residual stresses resulting from elevated temperatures encountered during SOI wafer processing and manufacturing. This residual stress manifests in slightly curled cantilevers after release. The negative effect of beam curvature is exacerbated by the extremely large length to thickness ratios necessary for THz PA sensing. By measuring the beam’s radius of curvature, the internal stress gradient (Δ*σ*) is evaluated using Stoney’s equation:

(3) $$\frac{1}{R}=\frac{6\left(1-\nu \right)\Delta \sigma }{Ed^{2}}$$

where, *R* is the beam’s radius of curvature, *E* is Young’s Modulus, *ν* is Poisson’s ratio and d is beam thickness. Although Stoney’s equation is not typically used to evaluate stress gradients in individual MEMS devices, it is appropriate here because the devices in this study have millimeter length scales. For example, using *R* = 1.11 m, the radius of curvature of a fabricated cantilever, *E* = 169 GPa, the Young’s Modulus for silicon, *ν* = 0.064, the Poisson ratio corresponding to the crystal orientation of the SOI material and *d* = 10 μm, the thickness of the beam, the cantilevers resulted in internal stress gradients of approximately Δσ = 2.702 N/m [22].

Since a cantilever’s spring constant is directly related to beam width and also the cubic of the thickness to length ratio, maximum tip deflection and therefore high sensitivity are achieved by increasing beam length (*L*), decreasing width (*w*) or decreasing thickness (*h*). Reducing the thickness to length ratio has the greatest impact, due to the cubic relationship. Unfortunately, millimeter length cantilevers with sub-10 μm thicknesses are very fragile and extremely difficult to fabricate using standard microelectronics and MEMS processes. Based on this, 10 μm-thick beams were used in this study.

## 2. Results

In this section, we describe initial beam selection criteria based on finite element methods (FEM) modeling and simulation results. Next beam design (*i.e.*, filleted corners) and fabrication (*i.e.*, dopant redistribution) improvements are presented. Finally, THz PA results, using the improved cantilever sensor, are presented.

### 2.1. Beam Modeling

**Figure 1:**
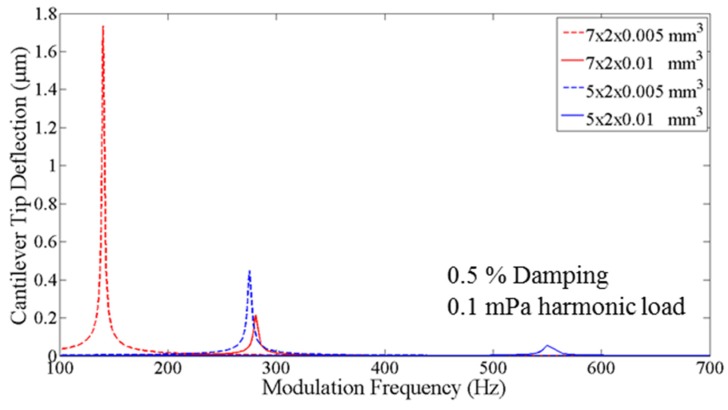
CoventorWare^®^ simulation results showing the effect of increasing cantilever length. In both the 5 μm thick beams and 10 μm thick beams, increasing the length from 5 mm to 7 mm resulted in approximately a 4 × increase in cantilever tip deflection and sensitivity.

Ideally, a long, thin cantilever would result in the highest PA sensitivity due to its lowered spring constant and high tip deflection. Figure 1 shows FEM beam deflection results when cantilever length and thickness were varied and an external 0.1 mPA harmonic load ranging from 100–600 Hz with 0.5% damping was applied. Note that this frequency range only exited the first order vibrating modes.

**Figure 2:**
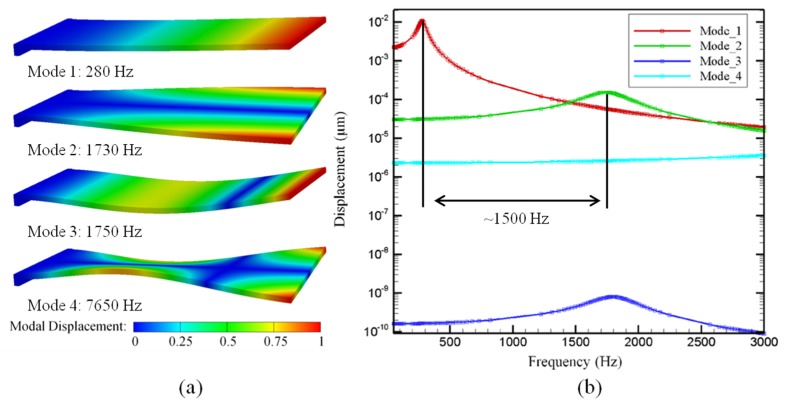
CoventorWare^®^ simulation results showing (**a**) first four modal harmonics of a simulated 7 × 2 × 0.01 mm^3^ cantilever and (**b**) the amount of tip deflection that occurs at these frequencies.

While longer cantilevers resulted in higher beam deflection and sensitivity, increasing the beam length also decreased the delta in modulation frequency between the first and second modal harmonics of the cantilever. Figure 2 shows the simulated structure and the resulting first four modal harmonics of a 7 × 2 × 0.01 mm^3^ cantilever.

As the length of a cantilever increases, the delta in frequency between the first two mechanical modes decreases, until it becomes difficult to vibrate the cantilever at resonance without immediately exciting higher order vibrational modes. Based on this, we focused our efforts on improving the device fabrication of a 7 × 2 × 0.01 mm^3^ cantilever beam sensor where the predicted difference between the first two higher modes was ~1500 Hz.

Figure 1 also shows that cantilevers with this geometry (7 × 2 × 0.01 mm^3^) had similar resonant tip deflections as the 5 × 2 × 0.005 mm^3^ geometry beams. The thinner, lower spring constant, beams, however, were flimsy and resulted in very low yield during fabrication. Consequently, this research effort focused on the thicker beams and enhancing overall system sensitivity by reducing internal beam stress, minimizing out of plane beam curvature, optimizing beam damping and by filleting the cantilever’s anchor and free end to minimize cracking and improve fabrication yield.

### 2.2. Dopant Redistribution

In the original fabrication process [19], the beam shape was defined by a front side deep reactive ion etch (DRIE), and the backside area, under the beam, was defined using DRIE all the way through the handle wafer up to the buried oxide layer resulting in a large open volume under the beam. The oxide was subsequently etched away using HF vapor to release the device. The resulting beams, however, contained some residual stress that caused slight out of plain curl after release. This residual stress (Δσ) in the cantilever, can be estimated using Equation (3).

**Figure 3:**
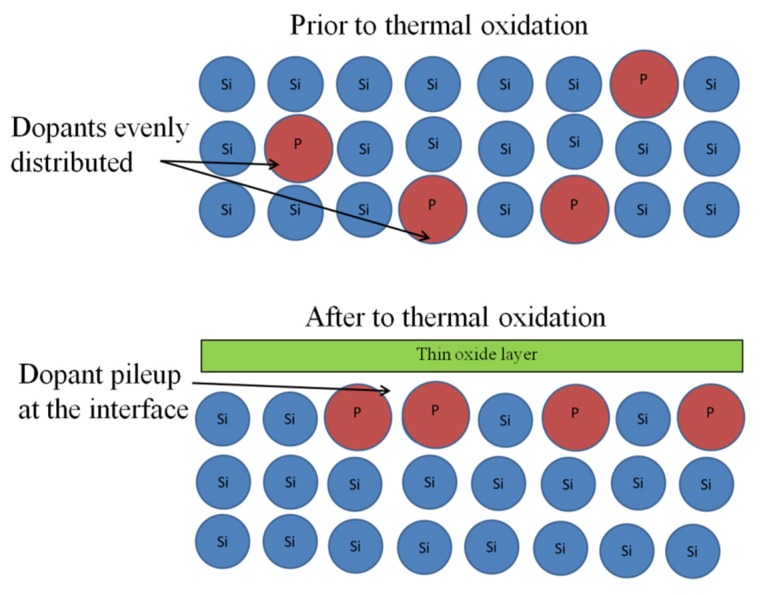
Illustration of phosophorous dopant atom redistribution and pile-up at the Si/SiO_2_ interface due to thermal oxidation.

In the improved fabrication process, a layer of thermal oxide was grown on the SOI wafers prior to starting device fabrication. This caused the phosphorous dopant atoms, in the n-type Si mechanical layer, to redistribute and pileup on Si side of the Si/SiO_2_ interface due to its relatively high segregation coefficient [23]. Additionally, the larger phosphorous atoms that gathered along the Si interface tended to reduce SOI mechanical layer stress due to improved bond alignment and atomic spacing. Figure 3 illustrates these concepts.

**Figure 4:**
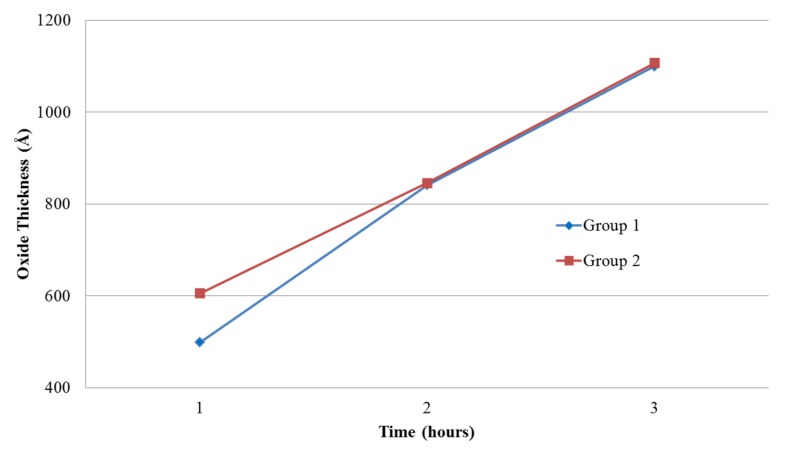
Plot of oxide thicknesses *versus* oxidation time for two data sets (*i.e.*, Groups 1 and 2) of three different oxide growth times. The samples were thermally oxidated in a tube furnace at 1000 °C for 1, 2 and 3 h, respectively.

In addition to redistributing phosphorous dopant atoms during the thermal oxidation, Si from the SOI mechanical layer was consumed at a rate of 44% during the SiO_2_ growth [23]. This consumption of Si altered the device layer thickness somewhat but since the thermals oxides were very thin, compared to the SOI mechanical layer, this effect was negligible. The thermal oxides were grown using H_2_O vapor in an oxidation furnace at 1000 °C for 1, 2 and 3 h. The resulting oxide thicknesses, measured using a Zygo™ (Middlefield, CT, USA) white light interferometer, exhibited linear thickness as a function of time as shown in Figure 4.

**Figure 5:**
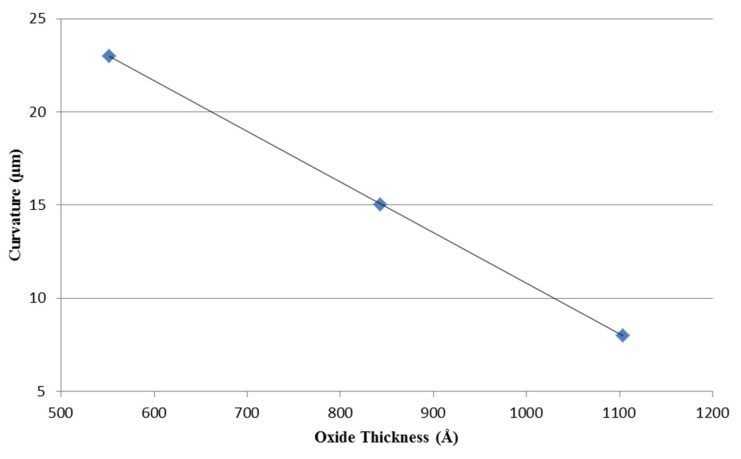
Plot of cantilever curvatures *versus* oxide thickness. Beam curvature decrease linearly with increasing oxide thickness grown prior to cantilever sensor fabrication.

After fabrication, individual cantilever radius of curvature was measured and the resulting radii of curvature were then plotted *versus* oxide thickness in Figure 5.

From Figure 5, the thicker thermal oxides resulted in reduced out of plane beam curvature (*i.e.*, flatter beams) after release due to lowered residual stress. Using Equation (3) and measured beam curvature, the results show that pre-oxide beams exhibit higher internal stress (Δ*σ*) than post-oxide beams. For example, the pre-oxide and post-oxide beams, in this work, resulted in Δ*σ* = 2.70 N/m and Δ*σ* = 1.10 N/m, respectively where the lower Δ*σ* directly correlated to lower beam curvature post release.

These results are due to the phosphorous dopant atoms being redistributed during the wet thermal oxide growth. The resulting flatter beams have a much reduced vertical gap distance along the beam’s length and at the beam’s end. This improved beam geometry and increased measured sensitivity combine to validate the reduced gas leakage during vibration assumption. In addition, because the system spring constant effectively increases, with less gas leakage, the sensor shows higher effective damping that manifests as increased sensitivity during ultra-low pressure gaseous species sensing.

### 2.3. Filleted Beam Corners

**Figure 6:**
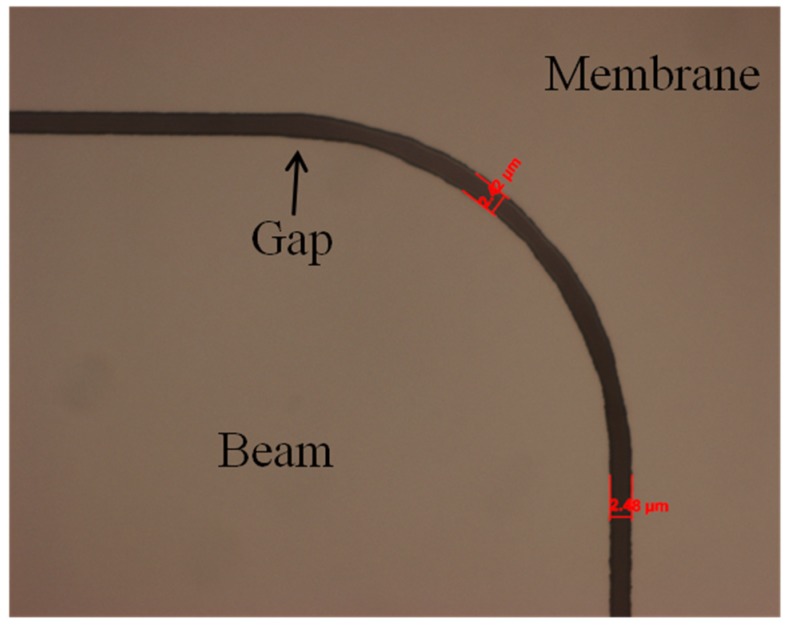
Filleted free end corner of a cantilever to reduce stress in the structure caused by having a sharp right angle. The ~3 μm gap was maintained throughout the corner and along the beam’s edge.

During fabrication and release, the beam corners (*i.e.*, free end and anchors) and the membrane area surrounding the cantilever were prone to fracture and cracking. This was, in part, due to the large surface areas (mm^2^) being only 10 μm thick. This was especially true of the area of silicon closest to the free end corner of the cantilever. The sharp right angle developed a high stress concentration that made the silicon prone to cracking during processing [24]. To counter this high stress concentration, the cantilever’s e free end and hinge corners were redesigned with fillets and then the beams were anisotropically dry etched using DRIE resulting in drastically improved fabrication yields. Figure 6 is an optical image of a filleted free end corner of a modified cantilever sensor.

The rounded or filleted beam anchors and corners markedly reduced the number of cracked beams and surrounding membranes during fabrication and release. The original beam design and fabrication process resulted in approximate 8.3% undamaged beams during the first six fabrication runs while the improved design and process yielded approximately 58.3% uncracked, useable beams during six additional fabrication runs.

### 2.4. THz PA Testing Results

**Figure 7:**
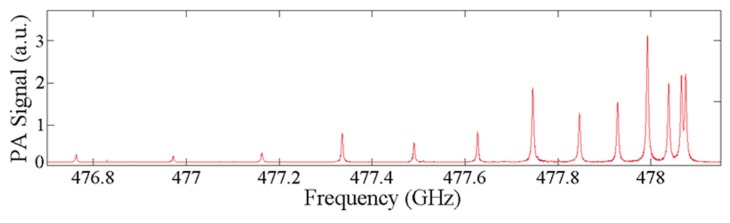
Methyl cyanide spectra data collected, using an improved 7 × 2 × 0.01 mm^3^ cantilever sensor design, at 15 mTorr.

Next, the improved cantilever beams were tested in a custom, low-volume (4 cm^3^), THz PA test chamber [19]. Figure 7 is a plot of methyl cyanide spectra data collected at 15 mTorr.

This spectral data, collected with a cantilever with filleted corners and a thermal oxide grown for 3 h, were collected using a 0.2 MHz step size with a 4 s excitation and a signal averaging time of 0.5 s. The entire data collection took approximately 9 h and the chamber pressure rose by only approximately 5 mTorr. The relatively long data collections, typical of very low pressure THz PA testing, are a result of the cantilever’s exhibiting long excitation times due to their relatively slow response rates in ultra low vacuum test environments [19]. The data showed an *α_min_* of 1.71 × 10^−5^ cm^−1^, a SNR of 2012 and a NNEA of 4.28 × 10^−10^ cm^−1^·WHz^−1/2^ (*P*_0_ = 25 μW and T = 1 s).

## 3. Discussion

In this research effort, a custom fabricated THz photoacoustic spectroscopy system, using an improved cantilever sensor, achieved an unprecedented sensitivity of 1.71 × 10^−5^ cm^−1^ and a NNEA of 4.28 × 10^−10^ cm^−1^·WHz^−1/2^ at 15 mTorr chamber pressure. These results represent approximately a 70% improvement over our previously reported results (e.g., *α_min_* of 197 × 10^−5^ cm^−1^, a SNR of 1221 and a NNEA of 1.39 × 10^−9^ cm^−1^·WHz^−1/2^ (*P*_0_ = 25 μW and T = 0.5 s)) which were an order of magnitude greater than anything else found in the literature at that time [19].

In general, lowering the chamber pressure in our novel, low volume (4 cm^3^) test chamber, resulted in lower SNR, higher (less sensitive) system sensitivity (*α_min_*) and a lower PA signal strength. Ideally, this manifests in lower NNEA at lower pressures and higher PA signal (and higher NNEA) at higher chamber pressures. With our fixed, low volume system, however, the limiting factor is that the number of detectable molecules also decreases with chamber pressure. In addition, the lower chamber pressures (and lower molecule counts) reduces cantilever damping significantly resulting in lowered PA response thus requiring relatively long (*i.e.*, hours) data collection times. At the minimum detectable chamber pressure (μTorr range), the undamped cantilever sensor cannot detect any PA signal resulting in a near zero *α_peak_* peak. At slightly higher chamber pressures, however, the quality of the cantilever sensor can help improve system sensitivity and NNEA.

Future work includes fabricating higher length to thickness ratio (7 × 2 × 0.005 mm^3^) beams that incorporate the design and fabrication improvements discussed in this paper. Additionally, future work includes adding a piezoelectric layer onto the cantilever to improve data collection over the current external laser interferometric-based system. This would enable a further reduced footprint system, as well as, being one step closer to realizing a hand-held THz PA chemical sensing system.

## 4. Materials and Methods

In this section, we present details for the entire improved cantilever fabrication process followed by details of the THz PA measurements.

### 4.1. Improved Device Fabrication

**Figure 8:**
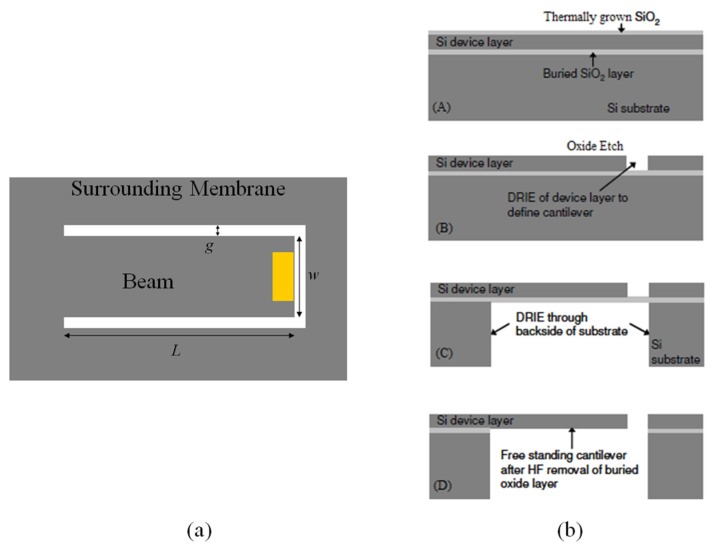
Cantilever sensor illustration showing: (**a**) a top view of the cantilever and the surround membrane area and (**b**) a cross sectional view of the individual fabrication steps where (A) is a wet thermal oxide grown at 1000 °C; (B) the oxide is etched away and the cantilever is shaped using deep reactive ion etching (DRIE); (C) is the backside DRIE handle wafer etch up to the buried oxide and (D) is the final oxide release etch using a hydrofluoric acid vapor etch.

The improved fabrication process begins by growing a thin thermal oxide layer, with H_2_O vapor in an oxidation furnace at 1000 °C, on n-type SOI wafers. Next, a thin layer (200/1000 Å) of titanium/Gold (Ti/Au) was evaporated onto the tip of the cantilever to serve as a reflective surface for interferometer measurements. The thin titanium layer acted as the adhesion layer for the gold. The SOI device layer was then patterned using a spin-coated positive photoresist to a thickness of approximately 1.8 μm. The resist was UV exposed for 8 s (total dose of 150 mJ/cm^2^) using a Karl Suss MJB3 mask aligner (Karl Suss, Garching, Germany). The exposed photoresist was developed for 30 s and hard baked for 5 min at 110 °C per manufacturer recommendations. Next, the samples were etched using DRIE to create a 3 μm gap in the device layer that defined the dimensions of the cantilever. As previously discussed, a uniform gap distance around the entire cantilever is critical and must be tightly controlled to minimize gas leakage between a vibrating cantilever sensor and the surrounding membrane. After the topside DRIE, to define the beam shape, a backside DRIE, under the beam, was etched through the entire handle wafer up to the buried oxide layer. This etch resulted in a large open volume under the beam. The thin oxide layer was subsequently etched, using HF vapor, to release the device. Figure 8 shows a top view of the cantilever sensor and a cross sectional view of the fabrication steps.

The compact, low-volume, test chamber and device test methodology are discussed next.

### 4.2. THz PA Testing Methodolgy

**Figure 9:**
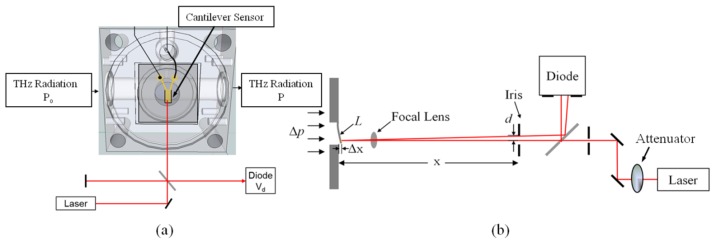
Schematic diagram (not to scale) showing (**a**) the custom, low-volume, photoacoustic (PA) test vacuum chamber with cantilever sensor position noted and (**b**) the PA optical measurement setup that was used to collect spectral data.

The compact, custom fabricated, THz photoacoustic chamber had overall dimensions of approximately 2 × 2 × 2 in^3^ and was constructed out of stainless steel [19]. The test chamber consisted of two segments; a front and back half with the cantilever sensor mounted in between them. The back portion of the chamber contained the absorption cell section while the front half had a small balance volume. The PA chamber and optics for the experimental setup were mounted on an optical bench. A HeNe (λ = 633 nm) laser beam, guided through a series of mirrors, beam splitter, irises, an attenuator, and focusing lenses, was reflected off the tip of the cantilever, back to a photodiode where the laser beam power was measured. An iris beam clipping method or optical beam deflection method, similar to Garcia-Valenzuela* et al.* [25] was used to collect the PA spectral data that was generated in test chamber. A schematic diagram of the PA cell, cantilever position and optical measurement is shown in Figure 9.

**Figure 10:**
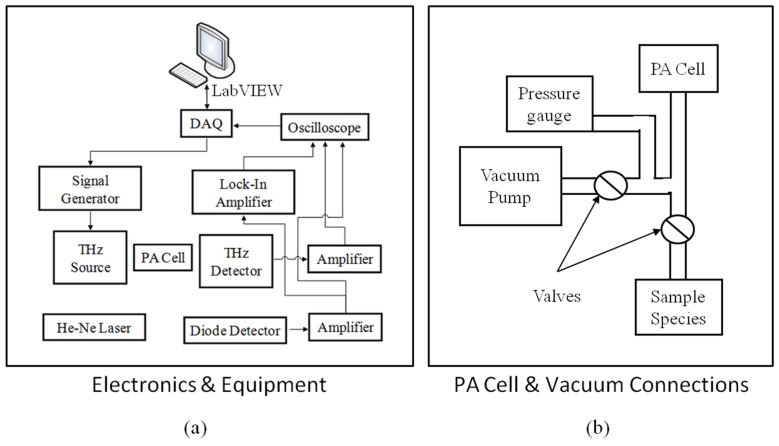
Block diagram showing (**a**) the electronics test equipment configuration and (**b**) the photoacoustic (PA) cell and test chamber vacuum connections.

To seal the chamber for ultra-low vacuum conditions, Teflon windows were used to enclose the ends of the absorption cell and an antireflective (AR) coated glass window sealed the balance volume so optical measurements of the cantilever deflection could be made with the HeNe laser. A Pfeiffer HiCube™ (Nashua, NH, USA) turbo pumping station was used to evacuate the chamber and achieve a low base pressure vacuum level. Liquid methyl cyanide (CH_3_CN) was introduced to the low vacuum environment through a series of valves and continuously monitored with a MKS Baratron^®^ capacitance monometer vacuum gauge (Cheshire, UK). Photoacoustic data collection was controlled using LabVIEW and the signals were collected with a National Instruments (NI) USB-6221 multifunction data acquisition (DAQ) card (Austin, TX, USA). To generate the THz radiation and cause the photoacoustic effect, a Virginia Diodes, Inc. (VDI, Charlottesville, VA, USA) diode and amplifier were used. The signal to the VDI THz radiation diode was provided by an Agilent E8254A PSG-A signal generator (Santa Clara, CA, USA). Controlled through the LabVIEW interface, the output of the signal generator was set to a specified THz radiation frequency that was amplitude modulated with a 50% duty cycle square wave at the desired modulation frequency. Emitted power by the THz VDI source ranged from 0.02–0.2 mW at 400 GHz. At low chamber pressures, the output power of the THz source was too high and therefore had to be attenuated at the low pressures to prevent molecular saturation. The photodiode signal was sent to a Stanford Research Systems SR560 preamplifier (Sunnyvale, CA, USA) and also to a SR530 lock-in amplifier (Sunnyvale, CA, USA). The signal from the Agilent E8254A signal generator was used as the reference signal for the SR530 lock-in amplifier (Santa Clara, CA, USA). The two channel output of the lock-in amplifier reported the magnitude and phase of the photodiode signal when compared to the reference signal. Figure 10 is a block diagram of the electronics and equipment used during PA spectroscopy.

For the iris beam clipping method, the measured photoacoustic signal at the diode was greatly affected by the iris placement at distance *x*, in front of the cantilever and the positioning of the focal lens. The focal lens was positioned such that the beam formed a tight spot at the tip of the cantilever and beam spot at the detector was smaller than the detector opening. Iris placement in front of the cantilever served two purposes. The first was to reduce the diameter of the incoming beam before the laser impinged on the cantilever. The second was to aperture the reflected light from the vibrating cantilever sensor before it reached the photodiode. The diameter of the iris closest to the chamber was set to ~0.8 mm. The final spot size of the HeNe laser beam at the photodiode was ~1.1 mm in diameter. Beam displacement, due to the PA effect, from steady state is expressed by

(4) $$d=x\text{ tan}\left(\sin^{-1}\frac{\Delta x}{L}\right)$$

where, *x* is the distance from the cantilever steady state position to the iris, Δ*x* is the cantilever deflection distance, and *L* is the cantilever length. In order to obtain the optimal signal, the THz source was modulated at the resonant frequency of the cantilever and the chamber, mounted on a three axis stage, was adjusted until the photodiode signal produced a symmetric amplitude sine wave. The sinusoidal peak-to-peak voltage signal from the photodiode and the magnitude of the lock-in amplifier signal were used to collect the photoacoustic spectral data from the sample gas. The peak-to-peak voltage signal created at the photodiode was due to the linear region of a three dimensional Gaussian beam profile caused by the clipping iris when the center of the beam was shifted by a total distance of 2*d* as shown on Figure 9.

## 5. Conclusions

This research effort achieved a sensitivity of 1.71 × 10^−5^ cm^−1^ and a NNEA of 4.28 × 10^−10^ cm^−1^·WHz^−1/2^ using a compact, low-volume THz photoacoustic spectroscopy and chemical sensing system. The achieved NNEA represents approximately a 70% improvement over previous results. Due to its compact size, the system could be used as a portable chemical sensing and spectroscopy platform. This would be a great advantage in comparison to a large traditional absorption cell for spectroscopy applications and may lead one day to a hand held THz chemical sensor or MEMS detector arrays for THz imaging applications.

## References

[1] West G.A., Barrett J.J., Siebert D.R., Reddy K.V. (1983). Photoacoustic spectroscopy. Rev. Sci. Instrum..

[2] Bell A.G. (1881). Upon the Production of Sound by Radiant Energy.

[3] Kosterev A.A., Bakhirkin Y.A., Curl R.F., Tittel F.K. (2002). Quartz-enhanced photoacoustic spectroscopy. Opt. Lett..

[4] Kosterev A.A., Tittel F.K., Serebryakov D.V., Malinovsky A.L., Morozov I.V. (2005). Applications of quartz tuning forks in spectroscopic gas sensing. Rev. Sci. Instrum..

[5] Liu K., Li J., Wang L., Tan T., Zhang W., Gao X., Chen W., Tittel F.K. (2009). Trace gas sensor based on quartz tuning fork enhanced laser photoacoustic spectroscopy. Appl. Phys. B.

[6] Borri S., Patimisco P., Sampaolo A., Beere H.E., Ritchie D.A., Vitiello M.S., Scamarcio G., Spagnolo V. (2013). Terahertz quartz enhanced photo-acoustic sensor. Appl. Phys. Lett..

[7] Krupnov A.F., Burenin A.V., Rao K.N. (1976). New methods in submillimeter microwave spectroscopy. Molecular Spectroscopy: Modern Research.

[8] Firebaugh S.L., Jensen K.F., Schmidt M.A. (2001). Miniaturization and integration of photoacoustic detection with a microfabricated chemical reactor system. J. Microelectromech. Syst..

[9] Ledermann N., Muralt P., Baborowski J., Forster M., Pellaux J. (2004). Piezoelectric Pb(Zr_x_, Ti_1−x_)O_3_ thin film cantilever and bridge acoustic sensors for miniaturized photoacoustic gas detectors. J. Micromech. Microeng..

[10] Kuusela T., Peura J., Matveev B.A., Remennyy M.A., Stus N.M. (2009). Photoacoustic gas detection using a cantilever microphone and III–V mid-IR LEDs. Vib. Spectrosc..

[11] Kuusela T., Kauppinen J. (2007). Photoacoustic gas analysis using interferometric cantilever microphone. Appl. Spectrosc. Rev..

[12] McNaghten E., Grant K., Parkes A., Martin P. (2012). Simultaneous detection of trace gases using multiplexed tunable diode lasers and a photoacoustic cell containing a cantilever microphone. Appl. Phys. B Lasers Opt..

[13] Adamson B.D., Sader J.E., Bieske E.J. (2009). Photoacoustic detection of gases using microcantilevers. J. Appl. Phys..

[14] Fonsen J., Koskinen V., Roth K., Kauppinen J. (2009). Dual cantilever enhanced photoacoustic detector with pulsed broadband IR-source. Vib. Spectrosc..

[15] Peltola J., Vainio M., Hieta T., Uotila J., Sinisalo S., Metsälä M., Siltanen M., Halonen L. (2013). High sensitivity trace gas detection by cantilever-enhanced photoacoustic spectroscopy using a mid-infrared continuous-wave optical parametric oscillator. Opt. Express.

[16] Kauppinen J., Wilcken K., Kauppinen I., Koskinen V. (2004). High sensitivity in gas analysis with photoacoustic detection. Microchem. J..

[17] Sievilä P., Chekurov N., Raittila J., Tittonen I. (2013). Sensitivity-improved silicon cantilever microphone for acousto-optical detection. Sens. Actuators A Phys..

[18] Koskinen V., Fonsen J., Roth K., Kauppinen J. (2008). Progress in cantilever enhanced photoacoustic spectroscopy. Vib. Spectrosc..

[19] Glauvitz N.E., Coutu R.A., Medvedev I.R., Petkie D.T. (2015). Terahertz Photoacoustic Spectroscopy Using an MEMS Cantilever Sensor. J. Micorelectromech. Syst..

[20] Pal P., Sato K. (2015). A comprehensive review on convex and concave corners in silicon bulk micromachining based on anisotropic wet chemical etching. Micro Nano Syst. Lett..

[21] Biswas K., Kal S. (2006). Etch characteristics of KOG, TMAH and dual doped TMAH for bulk micromachining of silicon. Microelectron. J..

[22] Boyd E.J., Uttamchandani D. (2012). Measurement of the anisotropy of young’s modulus in single-crystal silicon. Microelectromech. Syst. J..

[23] Jaeger R.C. (2002). Introduction to Microelectronic Fabrication.

[24] Dundurs J., Lee M.S. (1972). Stress concentration at a sharp edge in contact problems. J. Elast..

[25] Garcia-Valenzuela A., Villatoro J. (1998). Noise in optical measurements of cantilever deflections. J. Appl. Phys..

